# Malignant paraganglioma of the mesentery: a case report and review of literature

**DOI:** 10.1186/1477-7819-10-46

**Published:** 2012-02-23

**Authors:** Michael Chetrit, Pierre Dubé, Virginie Royal, Guy Leblanc, Lucas Sideris

**Affiliations:** 1 Departments of Surgery, Maisonneuve-Rosemont Hospital, University of Montreal, Montreal, Canada; 2 Pathology, Maisonneuve-Rosemont Hospital, University of Montreal, Montreal, Canada

## Abstract

Paragangliomas represent only 10% of chromaffin tissue tumors and those arising from the mesentery seem to be a rare occurrence. We report a case of a 55 year old man in whom an abdominal mass was discovered fortuitously by ultrasonography during a routine health exam. He presented occasional heart palpitations and diaphoresis as well as a well-demarcated mass upon abdominal physical examination. CT scan revealed a solid polylobulated mass in the right lower quadrant. Exploration laparotomy revealed a voluminous multi-nodular tumoral mass, which contained hemorrhagic spots. Histopathological studies confirmed the presence of a paraganglioma. The excision of the mass as well as the surrounding intestine and mesentery also revealed two lymphatic metastases, the first among 14 documented cases to be described concerning mesenteric paragangliomas. One year follow up and CT scan revealed neither recurrence nor the presence of distant metastases.

## Background

With an annual incidence estimated at 1/100,000, paragangliomas represent ten percent of catecholamine secreting tumors. Paragangliomas arise from chromaffin tissue most commonly found in the Zuckerkandl body, the sympathetic plexus of the urinary bladder, the kidneys, and the heart or in the sympathetic ganglia of the head or neck [1]. Some paragangliomas have been described in the Gastrointestinal System, the majority of which were associated with the duodenum. Only a very select few were described to arise from the mesentery [2]. Given their catecholamine secreting properties, paragangliomas have the potential to present as a mass with paroxystic symptoms of palpitations, pallor, tremor, headache and diaphoresis as well as hypertension [3]. This however is only the case in 25% of the paragangliomas. The rest have presented as abdominal masses with or without hypertension [2]. After a thorough review of the literature, this case report represents only the 14th case of a mesenteric paraganglioma and the first to document lymph node metastasis, a seemingly rare and unusual occurrence [Table 1].

**Table 1:** Documented data from 9 previous cases of mesenteric paragangliomas

NO of case	First Author	Year	Age/Sex	Symptoms	Size (cm)	Preoperative Diagnosis	Surgical Procedures	Mets	Follow up
1	Arean [11]	1956	32/M	Abdominal mass Nausea Vomiting Diarrhea	10	Abdominal Mass	Resection of intestine and mesentery along with mass	0	ND

2	Carmichael [12]	1970	62/F	Abdominal mass Hypertension	3.2	Abdominal Mass	Resection of intestine and mesentery along with mass	0	ND

3	Tanaka [13]	1991	29/F	Nausea Vomiting	10 × 9 × 7	Retroperitoneal mass	Resection of the mass	Liver	Alive

4	Onoue [14]	1999	38/F	Free	4.5 × 3.2 × 3.0	Mesenteric Mass HCC	Resection of intestine and mesentery along with mass Liver resection	0	Alive

5	Jaffer [5]	2002	76/M	Hypertension	8.5 × 8.0 × 2.0	Abdominal Mass	Resection of intestine and mesentery along with mass	0	ND

6	Muzaffar [2]	2002	76/M	Abdominal Mass	20 × 15	Abdominal Mass	ND	0	Alive

7	Kudoh [4]	2004	72/F	Abdominal pain and mass	10 × 10 × 9	Mesenteric tumor	Resection of intestine and mesentery along with mass	0	Alive

8	Svajdler [15]	2007	65/M		10 × 8			0	Alive

9	Na Guo [7]	2009	22/F	None	11.5 × 6.0 × 11.5		Resection of mass	0	Alive

10	Present Case	2011	55/M	Abdominal Mass	11.5 × 9.5 × 6.5		Resection of intestine and mesentery along with mass	2 LN	Alive, 56M

## Case presentation

**Figure 1:**
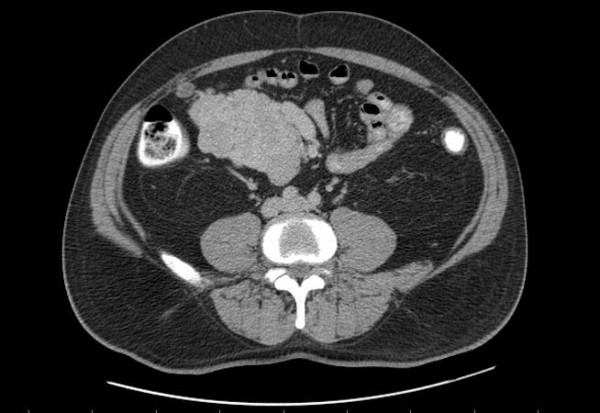
**Contrast Enhanced Tomography of the abdominal mass under investigation**. Contrast enhanced computer tomography scan showed a polylobulated mass measuring 10.1 × 7 cm in right hemiabdomen and right lower quadrant.

**Figure 2:**
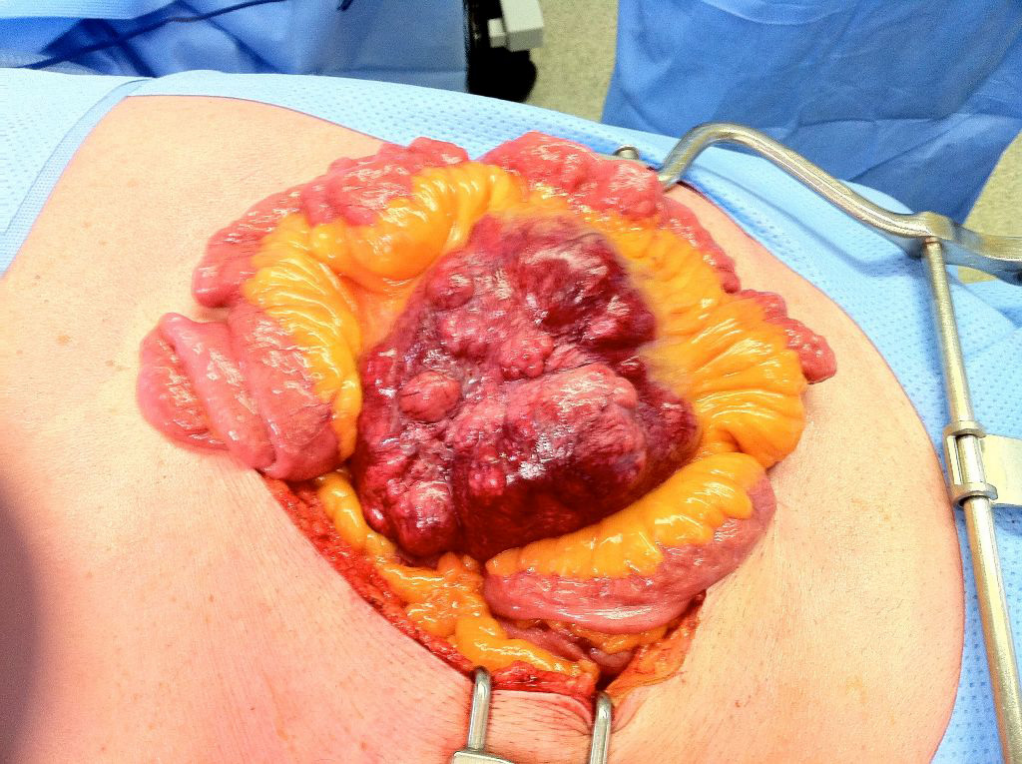
**Abdominal Mass In Vivo**. Multinodular Abdomen Mass with hemorrhagic spots in the anterior mesentery of the small intestines.

A 55-year-old man followed and treated for acid reflux and dyslipidemia as well as resection of a cutaneous sebaceous cyst, was admitted in our institution for the investigation of an abdominal mass discovered fortuitously during a routine annual abdominal ultrasound. The ultrasound, which revealed an asymptomatic hypoechogenous polylobulated solid mass in the right supraumbilical territory, was followed up and confirmed with a CT scan (Figure 1). The mass was also evident upon palpation of the right flanc, which revealed a well circumscribed mass measuring roughly 10 cm in diameter. Since a percutaneous biopsy was not possible, the patient underwent an exploratory laparotomy with resection of the mass (Figure 2) in question along with a segment of the small intestine followed by primary anastomosis. The post-operative course was uneventful.

The resected specimen consisted of segment of small intestine measuring 30 cm in length, accompanied by a small portion of adipo-fibrous mesenteric tissue. With the mesenteric tissue was a voluminous multi-nodular tumoral mass, measuring 11.5 × 9.5 × 6.5 cm. Upon further dissection of the solid mass, hemorrhagic spots were uncovered, which were well demarcated in the periphery.

**Figure 3:**
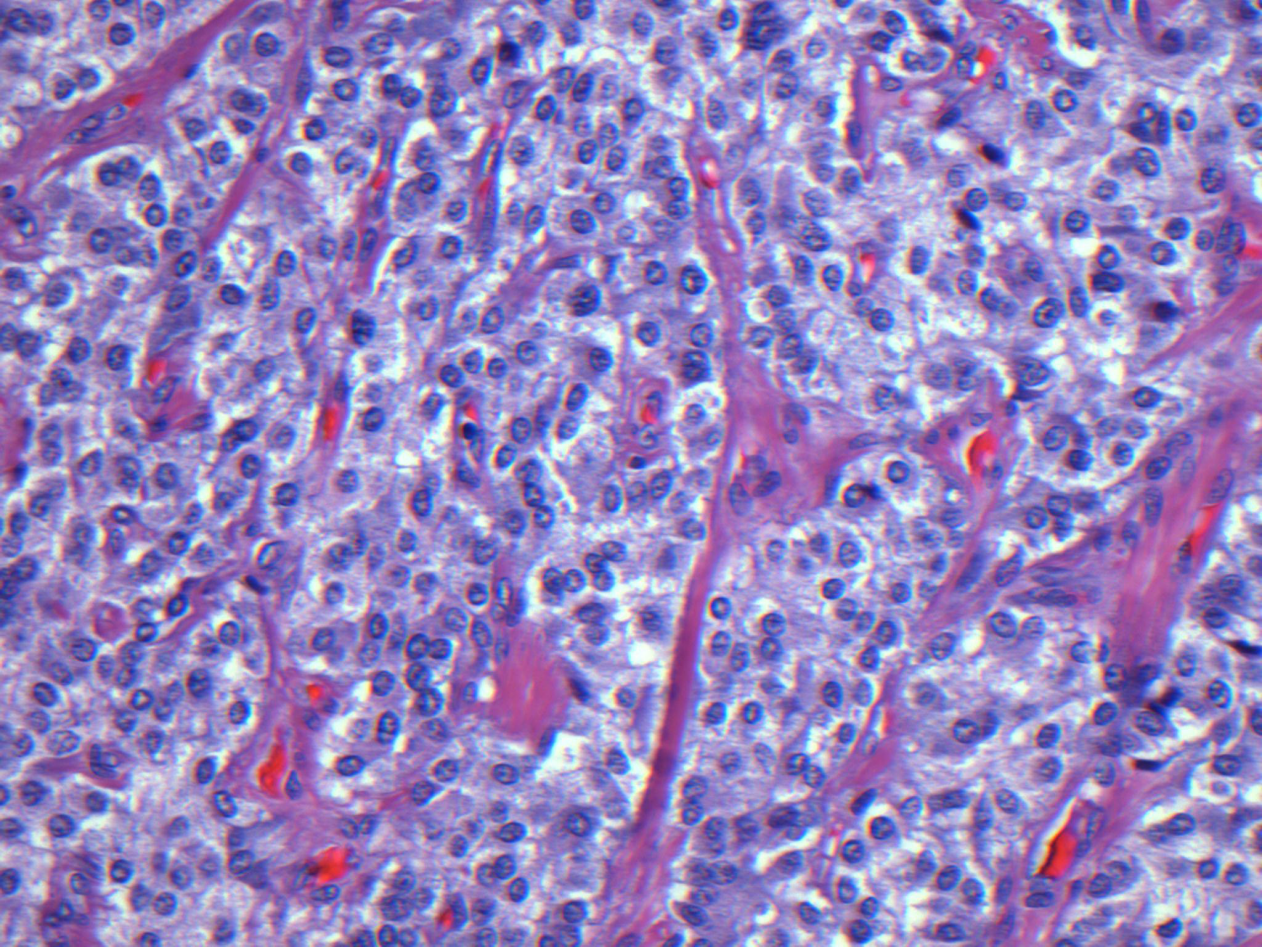
**Haematoxyin and Eosin staining of the resected specimen**. Groups of cells were separated by fibrovascular septae, giving a characteristic "zellballen" nested appearance. The group of cells were composed of polyhedral cells with granular amphopilic cytoplasm.

**Figure 4:**
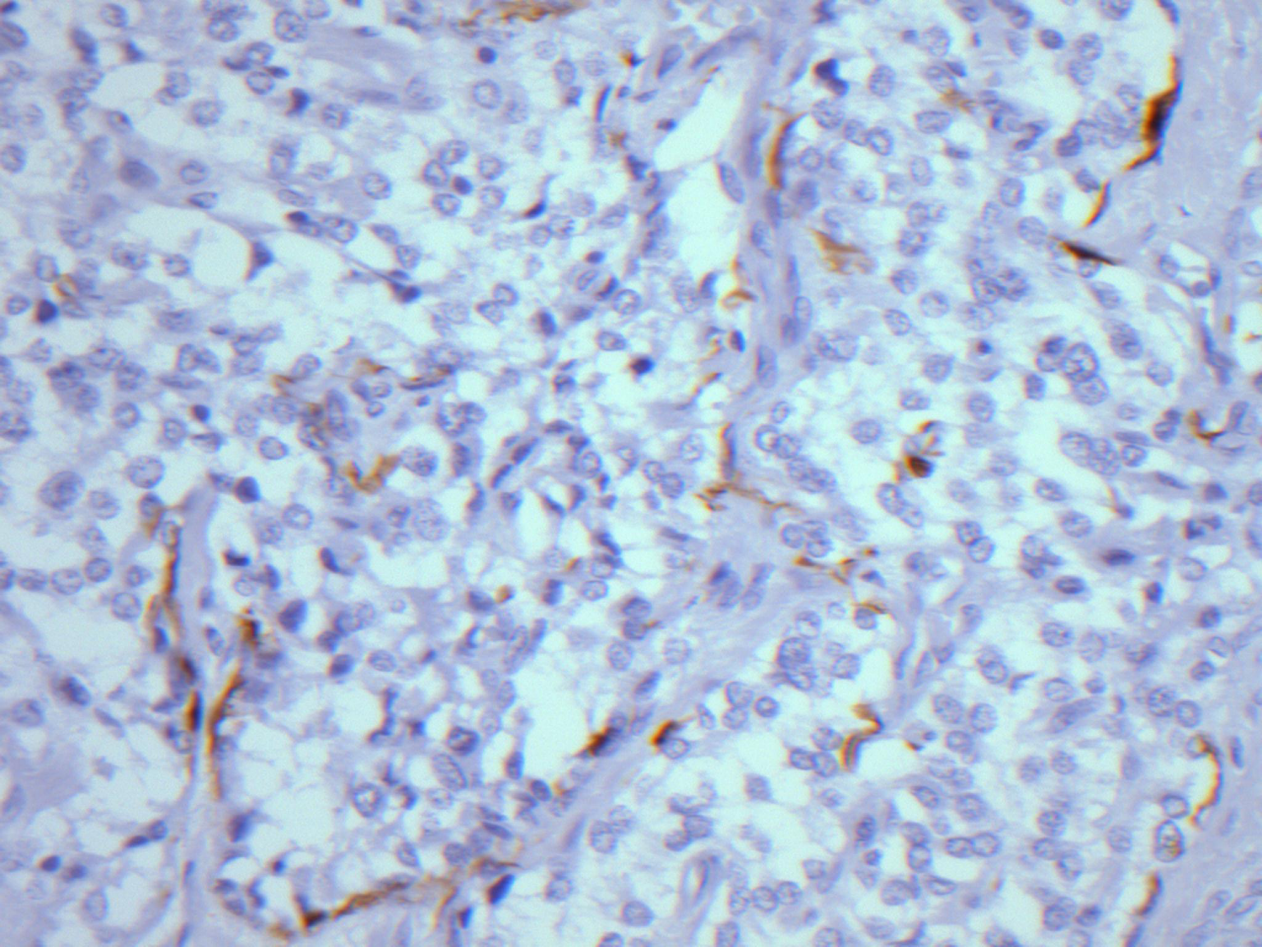
**Immunohistochemical staining of the resected specimen**. Immunohistochemical staining positive for protein S100 demonstrating sustentacular cells around the periphery of the cell nests and supporting the diagnosis of paraganglioma.

Histologic examination revealed a solid cellular proliferation with an alveolar and focally diffuse architecture. Groups of cells were separated by fibrovascular septae, giving a characteristic "zellballen" nested appearance. The group of cells were composed of polyhedral cells with granular amphophilic cytoplasm. Nuclei were round to oval in appearance with moderate atypia and prominent nucleoli. The mitotic count and proliferative indices were low (< 3/high power field and < 2%, respectively). (Figure 3) No vascular invasion or necrosis was noted. Nevertheless, local invasion was present with extension into peripheral adipose tissue and two metastases out of 9 resected lymph nodes were identified. Immunohistochemical staining was positive for chromogranin A, as well as for protein S100 demonstrating sustentacular cells around the periphery of the cell nests. Based on histologic and immunohistochemical features, a diagnosis of metastatic paraganglioma was proposed (Figure 4).

Given the presence of a paraganglioma, a urinary search for catecholamines and its derivatives was conducted and was unremarkable post operatively.

Throughout the procedure, and well before the identification of the mass, the patient remained asymptomatic aside from occasional palpitations and diaphoresis. The patient has been followed for almost a year and a follow up abdominal scan was conducted, all indicating the patient was free from any signs of recurrence including the palpitations and diaphoresis.

## Discussion

Neural crest cells give rise to the parenchymal cells of the paraganglia and other elements of the autonomic nervous system. These neural crest cells have the ability to migrate to various regions along the paravertebral and para-aortic axis, while remaining in close relation to the sympathetic nervous system. They extend to various places, anywhere from the neck to the base of the pelvis [4]. In rare occasions, paragangliomas have been identified in areas where chromaffin tissue has not yet been characterized, such as the genitourinary tract, spermatic cord, sacro-coccygeal area, anus, renal capsule, broad ligament, ovary and vaginal wall and can only be explained by the migratory property of the neural crest cells [5]. It has been hypothesized that tumors such as the one presented in this case report derive from the mesenteric paraganglionic tissue, which arises from vertebral migration from the root of the superior mesenteric artery [4].

Diagnosing paragangliomas, and in particular those of the mesentery, can be achieved via biochemistry and/or imagery. Given the capacity of a paraganglioma to secrete catecholamines, plasma or urinary metanephrines have been described in the literature as a very sensitive technique. Unfortunately the secreting property is only found in 25% of mesenteric paragangliomas [1]. Anatomical imagery with US/CT/MRI are equally as effective in identifying these abdominal masses. In addition, specific functional imaging with metaiodo-benzylguanidine scintigraphy or PET imaging with 6 [^18^F] fluoro-DOPA help identify and characterize the extent of the mass as well as the staging [6]. These techniques are then followed up with seemingly essential laparoscopic exploration and biopsy [7]. Finally, tumor resection is the form of treatment that has achieved the best results. Throughout all the cases described in the literature, none described any recurrence post-excision of the mass, but median follow-up was relatively short [Table 1]. Chemotherapy and radiotherapy have not demonstrated convincing results for patients with unresectable or metastatic disease. Its involvement remains palliative, as there is no current evidence of increased survival using these modalities [6]. Treatment with radiolabelled MIBG is gaining popularity given its avidity for the chromaffin cell tumors and in particular their metastases [8]. The literature stipulates that while ^131^I-MIBG is not a curative therapy, its involvement as an adjuvant to surgical resection as well as the possibility of a synergistic effect with chemotherapy seem promising and are venues to be explored in the near future [9]. Radioactive somatostatin analogues is yet another radiopharmaceutical to be considered [6]. Focus has now shifted to specific molecular targets involved in the malignant transformation of chromaffin cell tumors, and its development has shown signs of promise, yet development in these areas is still necessary [10].

With the exception of a single case documenting liver metastases, and to the best of our knowledge, this is the first case documenting regional lymph node metastases. As a result, this case can further be classified as one of malignant paraganglioma. This comes to no surprise given malignant chromaffin cell tumor have been documented to metastasize to local lymph nodes, bone liver and lung [6].

## Conclusion

Paragangliomas represent only 10% of chromaffin tissue tumors and the mesenteric form seems to be a rare occurrence, but should be among the preoperative differential diagnosis of abdominal masses of unknown etiology. The diagnosis is best achieved by imagery and laparoscopic exploration. Metastases although extremely rare, must be taken into account. Finally resection has proven to be the most effective method of treatment, while chemotherapy and radiotherapy have both been proven ineffective in previous cases.

## Consent

Written informed consent was obtained from the patient for publication of the Case report and any accompanying images. A copy of the written consent is available for review by the Editor in Chief of this journal.

## Competing interests

The authors declare that they have no competing interests.

## Authors' contributions

MC was involved in the review of literature, acquisition of data and drafting and completing the manuscript. LS conceived the study, participated in the coordination and acquisition of data and helped to draft the manuscript. VR carried out the immunohistological staining and interpretation of the resected specimens as well as contributed to the drafting of the manuscript. GL and PD participated in the critical review of the paper. All authors read and approved the final manuscript.
